# Case Report: Long-term survival of a patient with advanced rectal cancer and multiple pelvic recurrences after seven surgeries

**DOI:** 10.3389/fonc.2023.1169616

**Published:** 2023-05-15

**Authors:** Ye Ouyang, Yilin Zhu, Haoyi Chen, Guoquan Li, Xiongwei Hu, Hongyu Luo, Zhou Li, Shuai Han

**Affiliations:** ^1^ The Second School of Clinical Medicine, Southern Medical University, Guangzhou, China; ^2^ Department of General Surgery, Guangdong Province Huizhou Sixth Hospital, Huizhou, China; ^3^ Department of Gastrointestinal Surgery, General Surgery Center, Zhujiang Hospital, Southern Medical University, Guangzhou, China

**Keywords:** rectal cancer, recurrence, pelvic exenteration, long-term survival, KRAS mutation

## Abstract

**Background:**

Rectal cancer has a high risk of recurrence and metastasis, with median survival ranging from 24 months to 36 months. K-RAS mutation is a predictor of poor prognosis in rectal cancer. Advanced rectal cancer can be stopped in its tracks by pelvic exenteration.

**Case summary:**

A 51-year-old woman was diagnosed with advanced rectal cancer (pT4bN2aM1b, stage IV) with the KRAS G12D mutation due to a change in bowel habits. The patient had experienced repeated recurrences of rectal cancer after initial radical resection, and the tumor had invaded the ovaries, sacrum, bladder, vagina and anus. Since the onset of the disease, the patient had undergone a total of seven surgeries and long-term FOLFIRI- or XELOX-based chemotherapy regimens, with the targeted agents bevacizumab and regorafenib. Fortunately, the patient was able to achieve intraoperative R0 resection in almost all surgical procedures and achieve tumor-free survival after pelvic exenteration. The patient has been alive for 86 months since her diagnosis.

**Conclusions:**

Patients with advanced rectal cancer can achieve long-term survival through active multidisciplinary management and R0 surgery.

## 1 Introduction

According to the latest global cancer statistics, colorectal cancer (CRC) is the third most common type of cancer worldwide and the second leading cause of cancer-related deaths (1, 2). The number of CRC cases has been rising steadily in recent years, and there is a trend towards an earlier age of onset (3, 4). Patients with early CRC lack typical symptoms. Most CRCs are at an advanced stage when patients present with changes in bowel habits, blood in the stool, anemia or abdominal pain (5). In addition, 20% of CRC patients already have metastases at the time of diagnosis, with the liver, lung, peritoneum, and local lymph nodes being the most common metastatic sites (6, 7). The prognosis of metastatic CRC is poor, with a 5-year survival rate of only 12% (8). As newly developed chemotherapeutic agents and targeted drugs continue to be put into clinical use, coupled with the continued maturation of multidisciplinary oncology treatment, new hope has been brought to extend the overall survival(OS) of patients with advanced metastatic CRC (2). Rectal cancer accounts for approximately 30% of all CRCs and is one of the most common cancers of the gastrointestinal tract (9). Herein, we introduce a female patient with advanced rectal cancer who experienced multiple tumor recurrences, metastases, and underwent seven surgeries, and has survived for up to 86 months as of today.

## 2 Case presentation

### 2.1 Patient information and clinical findings

In October 2015, a 51-year-old lady was admitted to Zhujiang Hospital because of a change in her bowel habits that had been going on for more than six months. Six months ago, she noticed an increase in the frequency of her bowel movements, which were typically thin and unformed stools with two to three drops of blood after the stool, and she also had a feeling of anal drop before defecation, tenesmus and endless defecation. Thirteen years ago, she underwent a total hysterectomy for uterine fibroids. The patient had a history of diabetes mellitus and was advised to control her diet without taking medications. There were no other comorbidities. There was no history of alcohol or tobacco abuse and no family history of hereditary disorders. On examination, the patient was found to have tenderness pain in the lower abdomen, and rectal palpation revealed a mass of approximately 3×3 cm on the right posterior wall of the rectum 7 cm from the anus. There were no abnormal findings on physical examination of other systems.

### 2.2 Diagnostic assessments

**Figure 1:**
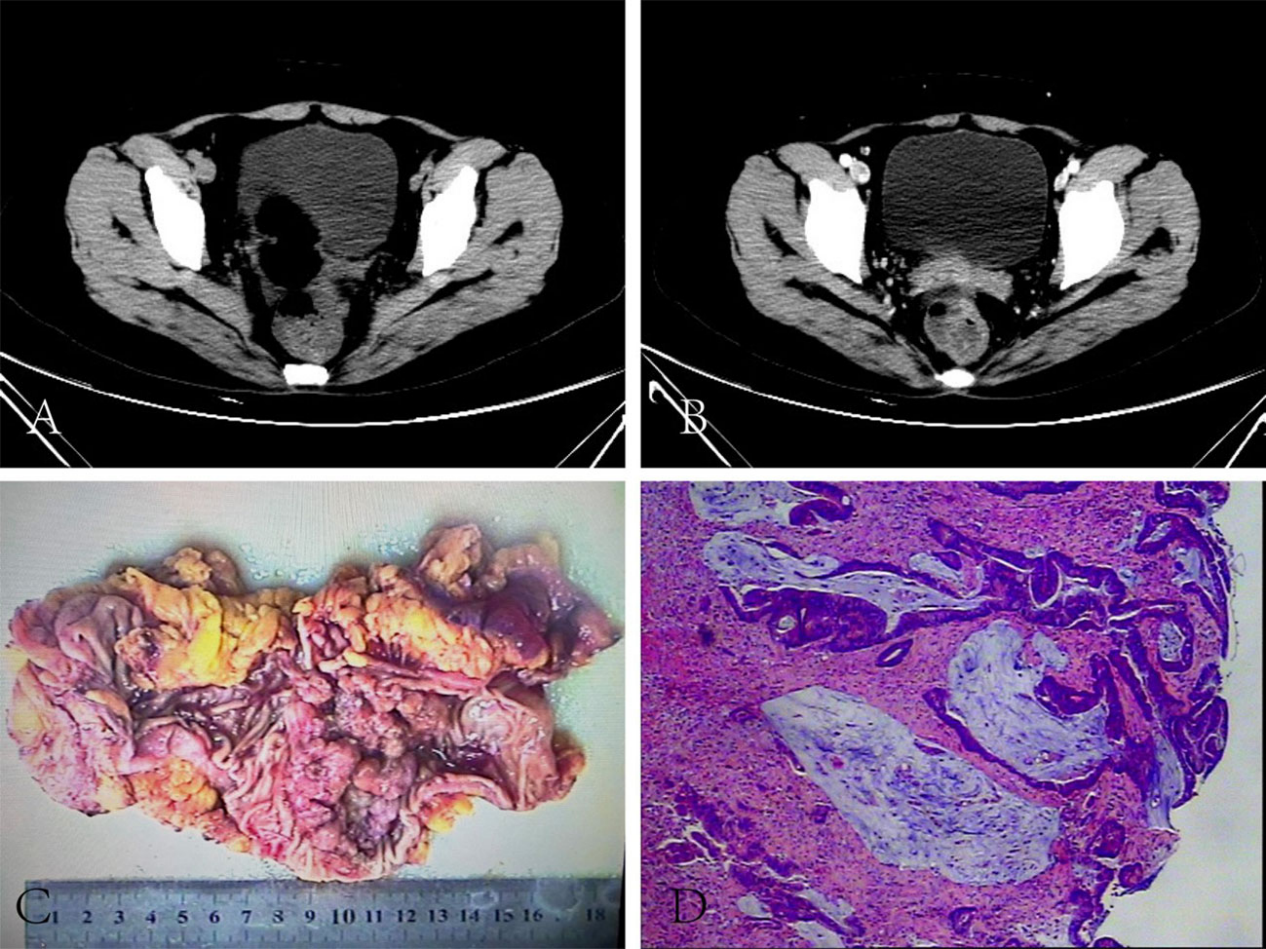
Preoperative CT and postoperative pathology for first radical rectal cancer: CT scan showed obvious thickening of the intestinal wall at the junction of the rectum and sigmoid colon, and soft tissue mass shadow with uneven density **(A)**. CT enhanced scan showed uneven enhancement of soft tissue masses at the rectosigmoid junction and local intestinal cavity was obviously narrow **(B)**. Part of intestinal duct (rectal mass), 18 cm long, 6-8 cm in circumference, 6cm away from the cut edge at one end, and a cauliflower-like mass 8 cm from the cut edge at the other end, 7cm x 5cm x 2 cm in size **(C)**. The cancer tissue of rectal mass was arranged in irregular glandular tubular and sieve shape, infiltrating the whole intestinal wall and breaking through the serous layer. The cancer cells were obviously heteromorphic, with large nuclei, deep staining and frequent mitotic images **(D)**.

A recent colonoscopy performed on the patient at the local hospital revealed a cauliflower-like mass located 7 cm from the anus, blocking the bowel lumen and restricting access to the colonoscope. Pathological analysis of the specimen indicated rectosigmoid junction cancer. The patient had elevated tumor markers, with blood levels of 85.2 μg/L for CEA and 82.1 kU/L for CA199. Body CT showed significant local bowel luminal narrowing, a soft tissue mass shadow with uneven density and enhancement, and significant thickening of the bowel wall at the junction of the rectum and sigmoid colon. Many enlarged lymph nodes were seen around this section of the bowel. The results of the abdominal CT and chest x-ray ruled out liver and lung metastases. According to the patient’s condition and her wishes, we performed a laparoscopic radical surgery for rectal cancer. Intraoperative exploration revealed a 5cm × 4 cm mass in the right wall of the rectum, which penetrated the plasma membrane layer and was strongly adherent to the right peritoneum and ovary. Two white metastatic cancer nodules could be seen at the top of the vagina. The right ovary and metastatic cancer nodules were also removed simultaneously with the consent of the family and with the assistance of the gynecology department. The postoperative pathology revealed a moderately differentiated adenocarcinoma of the rectum with a mucinous component, infiltrating the entire intestinal wall, invading the vasculature and nerves, with metastatic cancerous tissue visible in the mesenteric lymph nodes (4/14) and metastatic tissue from the right ovary. The patient’s final diagnosis was a moderately differentiated adenocarcinoma of the rectum (pT4bN2aM1b stage IV). (**Figure 1**) Immunohistochemistry showed CK (+), CDX-2 (+) and approximately 30% P53 in cancer cells. The patient had a microsatellite stable tumor microenvironment (TME) (MSS: MLH1 (+), MSH2 (+), PMS2 (+)) and Ki-67 approximately 40% (+). Genetic testing suggested mutations at the G12D locus of the KRAS gene.

### 2.3 Therapeutic interventions and follow-up

The first and second surgeries: In October 2015, the patient underwent laparoscopic radical surgery for rectal cancer and right ovariectomy. This was followed by six rounds of the XELOX (oxaliplatin and capecitabine) chemotherapy. However, 22 months after the surgery, the patient underwent a pelvic CT and plain and enhanced MRI at follow-up, which showed that the metastases had progressed and that there was a significantly larger mass anterior to the left external iliac artery than previously seen. In August 2017, laparoscopic exploration revealed a 3cm × 4cm irregular mass in front of the left iliac vessels, completely encapsulating the left ovary below, so the left ovary and metastases were removed. And six cycles of FOLFIRI (irinotecan, 5-FU, and calcium folinic acid) chemotherapy were started after the procedure.

The third surgery: At review in November 2019, imaging showed an occupying lesion on the right side of the anastomosis, poorly demarcated from the right wall of the vaginal stump, invading the surrounding tethered fascia and the right levator ani muscle, and tumor recurrence was considered. Once the diagnosis was clear, the patient was localized for radiotherapy and treated with local radiotherapy and a second-line chemotherapy regimen: FOLFIRI and bevacizumab. Due to 2 or 3 loose stools per day with painful bowel movements, the patient returned to hospital in March 2021. Since non-surgical treatment could not remove the lesion and prolong life, we performed a laparoscopic partial rectal resection and sigmoid colostomy for her.

The fourth surgery: In June 2021, a review revealed that the occupying lesion on the rectal stump continued to progress and increase in size, and tumor recurrence was considered. After multidisciplinary discussion, the final decision was made to perform abdominal radiofrequency ablation and particle implantation in collaboration with the interventional and radiology departments. The multidisciplinary consultation agreed that the patient’s lesion had progressed after previous first- and second-line treatment, and that she should be switched to third-line therapy with regorafenib, while TAC102 could be considered.

**Figure 2:**
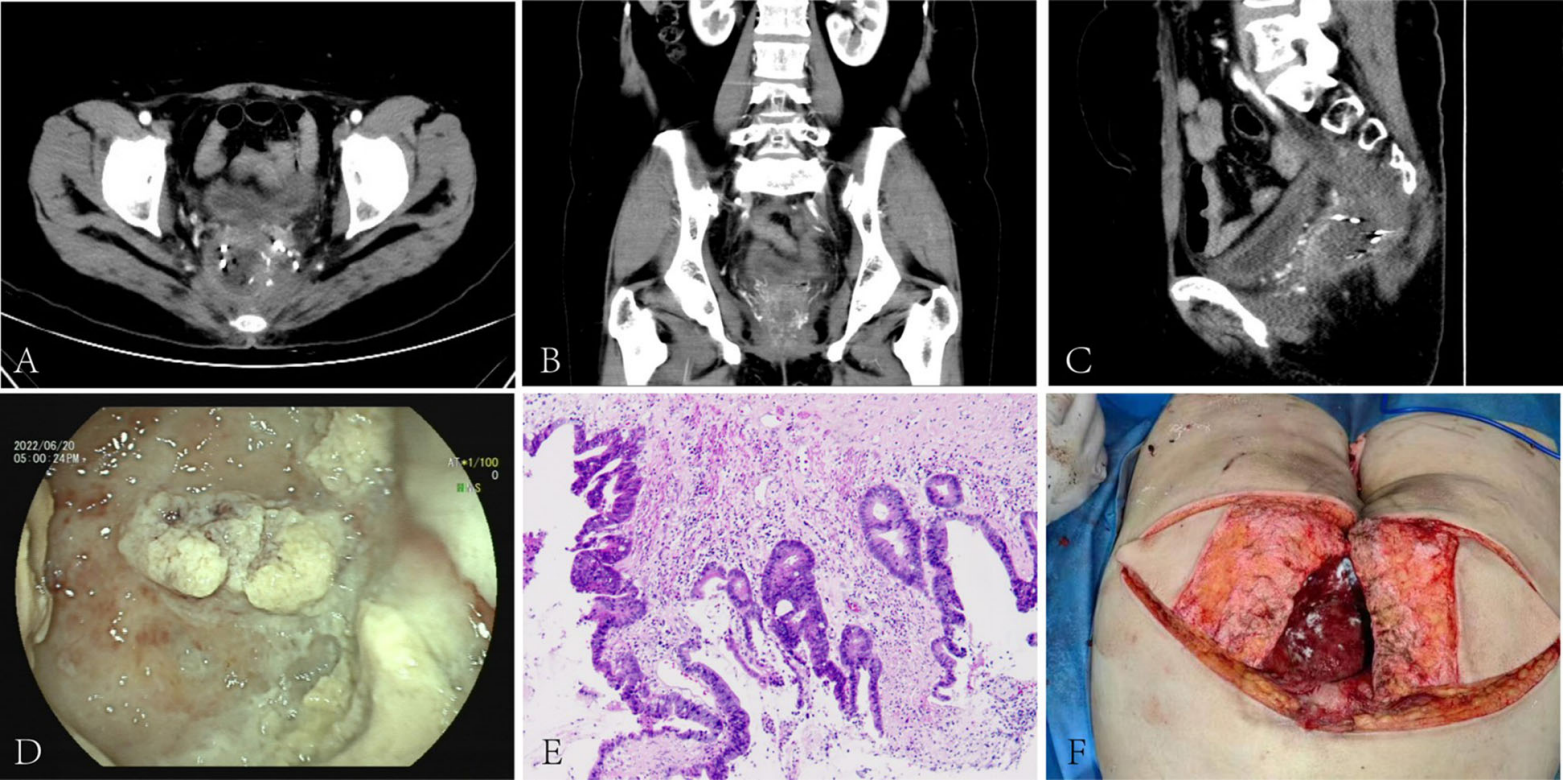
Pre-pelvic exenteration colonoscopy, CT, post-operative pathology and reconstruction: CT scan showed a mass-like slightly hypodense shadow next to the right side of the rectal stump. The lesion was poorly demarcated from the right vaginal wall and presacral soft tissue and invaded the surrounding tethered fascia and right levator ani muscle. The rectal cavity was compressed and deformed**(A-C)**. The blind end was observed at 8cm away from the anus through the natural anus, and the intestinal mucosa was covered with white contents**(D)**. The cancer tissue invaded the entire bowel wall, the entire vagina, involving the surrounding striated muscle tissue and adipose tissue, the local prolusion of the bladder mucosa, and localized necrosis of the serous layer and the muscular layer**(E)**. After pelvic exenteration, the patient underwent free myocutaneous flap graft **(F)**.

**Figure 3:**
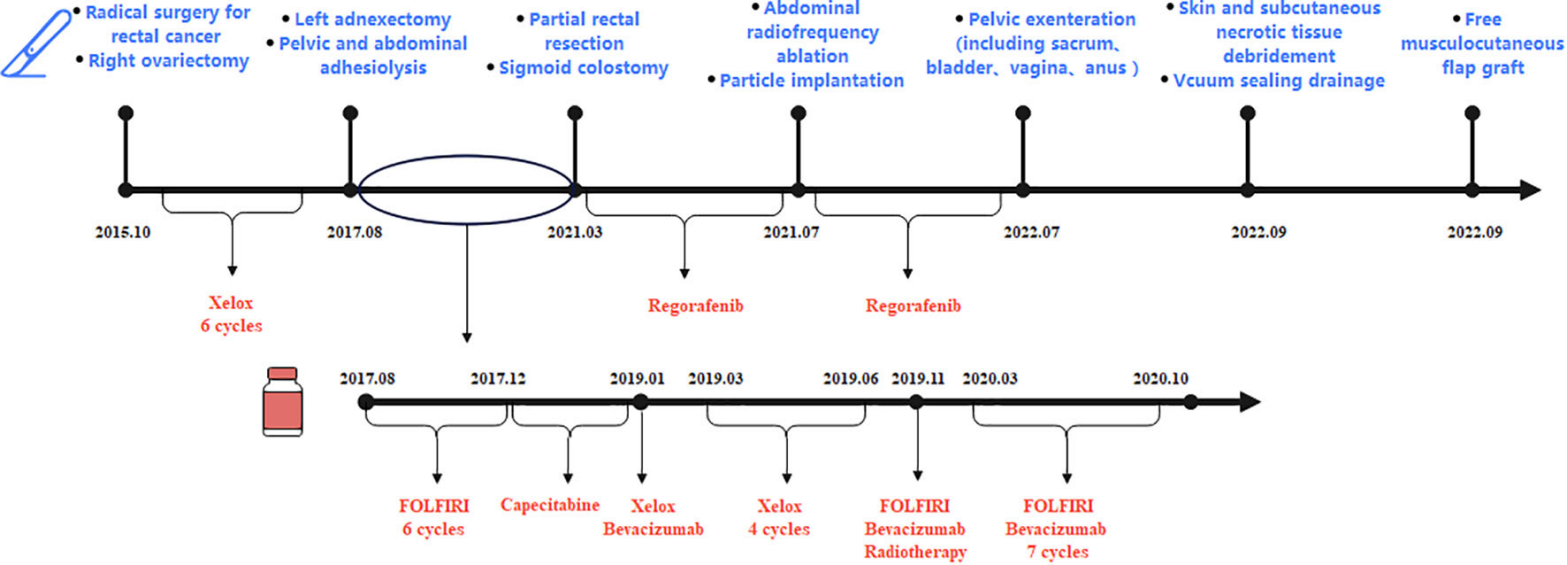
The patient had undergone seven surgeries (radical rectal cancer surgery and right ovariectomy in October 2015, left ovariectomy in August 2017, partial rectal resection in March 2021, radiofrequency ablation and particle implantation in July 2021, pelvic exenteration in July 2022, and two reconstructive surgeries in September 2022). During treatment, the patient had been taking chemotherapy drugs, including first-line chemotherapy drugs: XELOX (oxaliplatin and capecitabine), second-line chemotherapy drugs: FOLFIRI (irinotecan, leucovorin, and fluorouracil) and bevacizumab, and third-line therapy drugs: regorafenib.

The fifth to seventh surgeries: At the follow-up in July 2022, a rectal stump mass was again observed, together with an extended invasive lesion on the right side of rectal stump. Considering that almost all medical drugs had failed to control the disease, and radiotherapy and particle implantation had failed to control the tumor progression, we proposed pelvic exenteration to the patient after discussion with the multidisciplinary team. We actively sought the patient’s opinion and her main concern was to remove the tumor and reduce the threat to her life. She was willing to undergo aggressive surgical treatment and accepted our proposal. Therefore, we performed pelvic exenteration (transsacral resection of rectum, anus, bladder, vagina and sacrum) for the patient. Due to the large size of the surgical wound which was difficult to heal on its own, we performed a free myocutaneous flap graft and skin and subcutaneous necrotic tissue debridement on the lower back in September 2022. (**Figures 2**, **3**).

By 2023-1, the patient had been diagnosed with advanced rectal cancer for more than 7 years, had undergone 7 surgeries, and had survived up to 86 months. The patient’s tumor markers were not elevated during regular post-operative follow-ups after pelvic exenteration. The patient’s psychological burden was greatly reduced when we informed her that the tumor was most likely to be completely removed. Although she lived with a fistula bag after surgery, she was able to live independently with little restriction in general physical activity at our follow-up. We carried out the FACT-C (Functional Assessment of Cancer Therapy-Colorectal) test for the patient and her quality of life was quite satisfactory.

## 3 Discussion

The patient was still quite young when she was diagnosed with rectal cancer at the age of 51. In recent years, early-onset colorectal cancer (EOCRC) has gained widespread attention due to an alarming increase in its morbidity. Recent data from European registry studies show that the incidence of CRC in patients aged 20-49 has risen sharply over the past 25 years (10, 11). It is estimated that by 2030, one in ten colon cancers and one in four rectal cancers will be diagnosed in people under the age of 50 (12). Although the high incidence of diseases in young people is often due to genetic factors, this does not explain the rapid increase in recent years, so potential factors may also include westernized diets, obesity, antibiotics, infections and changes in the gut microbiome (13). Several studies have shown that the clinical characteristics and biological behavior of EOCRC differ significantly from those of conventional CRC. More invasive pathological factors are found in EOCRC, including higher tumor grade, lymphovascular infiltration, perineural infiltration, and elevated serum CEA, and are associated with worse OS (14). Current guidelines in the United States recommend starting screening at the age of 50 for average-risk individuals, which may lead to delayed diagnosis in EOCRC (15). In a study of 1,514 patients with rectal cancer, the median time from symptom onset to treatment is 217 days for those younger than 50, compared with 29.5 days for those older than 50 (16). Delayed diagnosis may lead to the development of cancer to a more advanced stage with a poorer prognosis (17, 18). Therefore, it is still worth investigating whether the age of screening for CRC needs to be brought forward and when to start screening to get the most benefit.

Colorectal cancer is known to have a poor prognosis as the second leading cause of cancer-related death. Fewer than 15% of patients with stage IV CRC survive within 5 years of diagnosis (19), and the majority of patients with metastatic colorectal cancer currently have a survival of 24 to 36 months (2). This patient was diagnosed with stage IV rectal cancer at the time of detection, and several factors suggested that she would have a much shorter survival. Firstly, the patient had a K-RAS mutation. As the most common and first discovered mutation in colorectal cancer, KRAS mutations are seen in between 30% and 40% of cases and are often considered a poor prognostic indicator (20, 21). On one hand, KRAS mutant cancers are highly invasive (22). On the other hand, KRAS mutations have also been found to be an important predictor of lack of response to treatment with epidermal growth factor receptor monoclonal antibodies (23–25). In addition, studies have shown that KRAS G12D mutant tumors have a poorer response to chemotherapy and radiotherapy, and significantly shorter progression-free survival (PFS) and OS (26). The patient in our case report had the G12D mutation, which largely predicted her poor prognosis. Secondly, ovarian metastases were found during the treatment. Metastases from the gastrointestinal tract to the ovary are known as Krukenburg tumors, which are very rare metastatic malignancies of the ovary with an incidence of 0.16/100,000 (27). The prognosis for Krukenburg tumors is poor, with a median survival of 23 months for limited metastases and an average of 14 months for widespread metastases, regardless of the treatment method (28). Remarkably, the patient has lived for 86 months so far. Our analysis of her prolonged survival could be as follows:

First, despite numerous recurrences and involvement of nearby organs, the patient showed no evidence of liver or lung metastases. Metastases to secondary organs, such as the liver and lung, are thought to be a major contributor to colorectal cancer-related mortality (29). According to a review of 14 randomized clinical trials, median OS was 19.1 months for CRC patients with liver metastases and 24.6 months for those with lung metastases (30). The exemption of liver and lung is beneficial for patient survival to some extent.

Second, the only salvage procedure is pelvic exenteration (PE) when advanced rectal cancer has spread to many nearby organs and recurred frequently (31, 32). To achieve a tumor-free margin, PE is a procedure that completely eradicates all pelvic malignancies (33). Recent improvements in perioperative care, surgical technique, and multidisciplinary team approaches have reduced surgical mortality and increased access to therapy for rectal cancer whose recurrences and metastases are confined to the pelvis (34, 35). In this case, we removed the patient’s vagina, bladder, anus, rectum, and sacrum. The patient underwent two further reconstructive procedures, as research suggests that myocutaneous flap reconstruction and long-term pelvic/perineal drainage after PE may reduce infection rates and improve wound healing (36). Despite the risk associated with PE, most of these patients face a median OS of approximately 7 months if surgical resection is not attempted (37). Therefore, pelvic exenteration may be the only hope for long-term survival of rectal cancer patients without distant metastases (38).

Finally, the patient had many pelvic metastases and tumor recurrences that were surgically significant, and R0 resection was achieved in almost all surgical procedures. Margin status is the most important predictive factor for the outcome of pelvic exenteration for recurrent rectal cancer, according to a number of studies. The greatest survival benefit after PE is seen in patients with negative margins or R0 resection (39–42). The American Joint Committee on Cancer was the first organization to recognize the importance of the ‘R’ status of tumor resection in 1977 (43). Histopathological evaluation of R0 resection is considered to be a circumferential margin (CRM) of >1 mm.R1 resection is the presence of microscopic residual lesions defined as CRM ≤1 mm, while R2 resection is the presence of residual sarcoid lesions (39). R0 resection status remains the most important determinant of disease-free survival and OS in resectable rectal cancer. One study showed that the median survival was 43 months for patients with R0 resection, 21 months for patients with R1 resection, and 10 months for patients with R2 resection, and three-year survival rates were 56.4%, 29.6%, and 8.1% for R0, R1, and R2, respectively (39). In addition, multiple studies have suggested that R0 resection is associated with a good prognosis (39–41, 44). The patient we report achieved R0 resection at almost all surgeries of tumor recurrence or adjacent organ involvement and is still tumor free today.

There is currently no clinical consensus on the value of resection of distant metastases in the management of patients with advanced CRC. A number of studies have shown that surgical resection is the main treatment for patients with liver and lung metastases from CRC to achieve ‘no evidence of disease’ status and long-term survival (45–50). The Japanese guidelines for the treatment of CRC recommend surgical treatment of the metastatic lesion if the patient can tolerate it and both the primary colorectal and metastatic lesions are completely resectable (7). According to the results of the Bologna Multidisciplinary Rectal Cancer Group, people who underwent metastatic resection had a longer OS (51).. However, the main problem with resection of liver metastases is the high recurrence rate, which is as high as 75% within 2 years and is the main cause of postoperative mortality in patients (52). Other studies have shown that lung metastasectomy leads to a reduced quality of life and reduced health benefits for patients after surgery (53, 54). In addition, Riccardo Lemini et al. showed no difference in survival outcomes between patients who underwent metastasectomy and those who did not (55). Therefore, more studies are needed in the future to fully understand the role of metastasectomy in order to provide more effective treatment strategies for patients with advanced CRC who develop distant metastases.

The incidence of ovarian metastases from CRC ranges from 1.6% to 6.4% (56),with approximately 40-60% of patients with ovarian metastases presenting with bilateral involvement (57, 58). It has been shown that the ovary appears to be a ‘sanctuary’ for tumor cells, providing a favorable microenvironment for tumor growth, and that metastatic ovarian tumors do not respond well to chemotherapy. Therefore, surgery appears to be the only viable alternative (57, 59). In this case, we found involvement of the right ovary during the initial radical surgery for rectal cancer, and performed resection of the metastases at the same time, and then found metastases in the left ovary 2 years later and promptly resected them. This raises the controversial question of whether prophylactic removal of the other ovary is necessary in the case of metastases from one ovary. Bilateral ovaries are interconnected by branches of the fundic artery and CRC can develop bilateral ovarian metastases by a haematogenous route (60, 61). In addition, CRC may also increase the risk of ovarian metastases by spreading through the peritoneum (62). Utku Akgor et al. argue that: Microscopic metastases can occur even in normal-appearing ovaries, and bilateral salpingo-oophorectomy should be the standard of care for all patients with ovarian metastases from CRC (59, 63).

The standard treatment for colorectal cancer is surgical resection combined with adjuvant chemoradiotherapy (64). However, for the past 30 years, patients with KRAS mutations have been in a drug-free situation, because targeting KRAS-driven cancer therapy faces several major challenges: first, KRAS has a similar GTP/GDP-binding region to other RAS family members, making it difficult to achieve targeted therapy (65). Second, the high affinity of KRAS for GTP/GDP makes it difficult to find effective inhibitors (65). There are now new developments in targeting KRAS for the treatment of CRC. Several groups have reported the development of direct KRAS small molecule inhibitors that bind directly to KRAS and inhibit nucleotide exchange or block the interaction between RAS-GTP and SOS (66–69). In addition, RT11, a cytoplasmic penetrating antibody targeting RAS mutant tumors, has been shown in preclinical studies to overcome drug resistance when combined with cetuximab (70). In addition, immunotherapy has shown clinical promise in the treatment of gastrointestinal tumors and autologous T cell transplantation for the treatment of KRAS G12D CRC is expected to enter the clinical practice (71), which may lead us to bid farewell to the era of KRAS ‘incurable’.

## 4 Conclusion

Although colorectal cancer patients with KRAS mutations and multiple recurrences often have a poor prognosis, the patient in our reported case, who had advanced metastatic colorectal cancer with KRAS mutations, had an exceptionally long survival. This is due to a combination of factors, including vigorous medical and surgical treatment with intraoperative R0 resection, and the fact that the patient’s liver and lungs were free of tumor metastases. The patient achieves tumor-free survival with good quality of life after pelvic exenteration. Our diagnosis and treatment provide experience and hope for patients with multiple recurrences and local metastases of colorectal cancer.

## Data availability statement

The original contributions presented in the study are included in the article/supplementary material. Further inquiries can be directed to the corresponding authors.

## Ethics statement

The studies involving human participants were reviewed and approved by the Ethics Committee of Zhujiang Hospital. The patients/participants provided their written informed consent to participate in this study. Written informed consent was obtained from the participant/patient(s) for the publication of this case report.

## Author contributions

YO: literature research, manuscript preparation. YZ: literature research, manuscript preparation. HC: literature research, manuscript preparation. GL: literature research. XH: literature research. HL: manuscript final version approval. ZL: manuscript final version approval. SH: manuscript final version approval. All authors contributed to the article and approved the submitted version.
